# Peer support interventions in maternal and child healthcare delivery in sub-Saharan Africa: protocol for a realist review

**DOI:** 10.1186/s13643-023-02366-3

**Published:** 2023-10-25

**Authors:** Gordon Dugle, John Antwi, Wilm Quentin

**Affiliations:** 1 School of Business, SD Dombo University of Business and Integrated Development Studies, Box UY 36, Wa, Ghana; 2 School of Business, University for Development Studies, Post Office BOX 1350, Tamale, Ghana; 3 Department of Healthcare Management, Technische Universität Berlin, Berlin, Germany; 4 German West-African Centre of Global Health and Pandemic Prevention (G-WAC), Kwame Nkrumah University of Science and Technologies, Kumasi, Ghana

**Keywords:** Peer support, Maternal and child healthcare, Realist review, Complex intervention, Sub-Saharan Africa

## Abstract

**Background:**

Peer support has been proposed as a promising policy intervention for addressing adverse maternal and child healthcare (MCH) outcomes in sub-Saharan Africa (SSA). Existing reviews on peer support largely draw on evidence from high-income countries or focus on single services like breastfeeding, nutrition or postnatal care. In contrast, this review aims to provide a comprehensive overview of the empirical literature on peer support interventions across various MCH services in sub-Saharan Africa. Specifically, we aim to understand how, why, for whom, and in what circumstances different forms of MCH peer support interventions contribute to improving healthcare outcomes in sub-Saharan Africa.

**Methods:**

This review follows five iterative steps for undertaking realist reviews (1) defining the review scope; (2) developing initial programme theories; (3) searching for evidence; (4) selecting and appraising evidence; and (5) extracting, analysing and synthesising evidence. Four databases–Cochrane Library, PubMed, CINAHL, and EMBASE–were repeatedly searched between March and June 2021. From a large volume of records retrieved from the database and citation search, 61 papers have been selected for review. We will conduct a second search of the same database covering June 2021 to the present before the final extraction and synthesis. The final list of selected papers will be imported into NVivo 12 software and organised, extracted, analysed and synthesised iteratively to examine and illustrate the causal links between contexts, mechanisms and outcomes of MCH peer support interventions in SSA. We have drawn on the existing literature on peer support in healthcare generally to develop initial programme theories. We will then use the empirical literature on MCH peer support interventions in SSA, inputs from a stakeholders’ workshop in Ghana and a conference presentation to refine the initial programme theory.

**Discussion:**

The review will develop an explicit theory of peer support intervention in healthcare delivery and provide insights for developing evidence-informed policy on the intervention. Drawing lessons from the different national contexts and diverse areas of MCH in SSA, the review will provide an analytically generalizable programme theory that can guide intervention design and implementation. While focusing on MCH peer support interventions in SSA, the review contributes to evolving conversations on the use of theory for health policy planning and complex intervention design and implementation globally.

**Trial registration:**

PROSPERO registration ID: CRD42023427751.

**Supplementary Information:**

The online version contains supplementary material available at 10.1186/s13643-023-02366-3.

## Background

Adverse outcomes in maternal and child healthcare (MCH) remain critical national and global health priorities. Although most developed countries have made significant progress in MCH over the last two decades, much of the world’s adverse outcomes occur in Sub-Saharan Africa (SSA). Progress reports on the Millennium Development Goals (MDGs) and Sustainable Development Goals (SDGs) have consistently pointed to persistent adverse outcomes in SSA [1–4]. For instance, the World Health Organization (WHO) estimates that about 66% of the world’s maternal deaths occur in SSA [3], and under-five mortality in 2020 was twice as high in SSA (72 deaths per 1000 live births) than the global average [5].

Peer support has emerged as a policy intervention for promoting effective and sustainable MCH delivery over the last two decades. Peer support broadly refers to ongoing social and practical assistance provided by non-professionals to help people manage their health conditions, respond to particular healthcare needs, and/or contribute to overall well-being. It includes recurring interactions between people such as family members, neighbours, friends, or other associates but excludes incidental interactions and formal relationships like contacts between patients and service providers [6, 7]. Policy-makers and scholars have suggested that peer support is a promising intervention for promoting physical, mental and social well-being [8, 9].

Various peer support programmes in SSA have emerged to address maternal, newborn and child healthcare gaps. Examples include the Kenyan Mentor Mother Programme established in 2012 as part of the country’s national strategy for preventing mother-to-child transmission of HIV and syphilis. In addition, the mothers2mothers (M2M) programme, which started in South Africa in 2001, has quickly expanded to nine other SSA countries (namely, Angola, Ghana, Lesotho, Kenya, Malawi, Mozambique, Tanzania, Uganda and Zambia). M2M uses women living with HIV as social support and mentors to help HIV-positive pregnant women stay healthy and prevent mother-to-child transmissions.

While policy and scholarly interests in peer support in MCH are growing, there is less clarity on how, why, for whom, and in what circumstances peer support is effective (or not). Existing reviews on the effect of peer support in specific MCH areas like nutrition [10], breastfeeding [11–13], and postnatal care [14] have focused exclusively on high-income countries. Reviews that have included evidence from low- and middle-income countries have only evaluated the effect of peer support on (exclusive) breastfeeding [15–17]. However, MCH comprises a wide range of services for promoting health and avoiding morbidity and mortality of mothers (women of childbearing age), infants, children and adolescents [18]. It ranges from antenatal care for mothers and growth monitoring in children to sexual education for adolescents. Yet, no review has explored peer support across this diverse set of MCH services (e.g., breastfeeding, family planning, antenatal care and postnatal care). This limits our understanding of the processes by which peer support for different MCH services improves delivery outcomes across a range of national and local conditions.

Recent discussions in research and policy circles acknowledge that peer support interventions are context-bound programmes as they vary in terms of design, scope of activities and the broader social structures within which they are developed and implemented [8, 9, 14]. This understanding of peer support interventions as context-bound aligns closely with the realists’ position that interventions will work in certain contexts but not in others [11, 19]. Accordingly, we aim to undertake a realist review to examine and illustrate how, why, for whom, and in what circumstances different forms of peer support interventions contribute to improving MCH outcomes in SSA.

The review is intended as an initial step towards a transdisciplinary research project on developing, implementing and evaluating health-related peer support interventions. The project–still at a conceptual stage–aims to foster transformational advances in the generation and use of research and evidence to inform the development and implementation of peer support interventions in healthcare across national, regional and global levels. The project will be led by this review team together with other academics and practitioners from diverse disciplinary backgrounds involved in developing and implementing peer support interventions in healthcare. The findings of this review will inform the next stage of the project.

## Methods

### Realist review

Realist review or synthesis (used here interchangeably) is a theory-driven method of synthesising evidence and is rooted in the realist philosophy [20–22]. Conducting a realist review of evidence about a phenomenon (in this case MCH peer support interventions in SSA) involves addressing the general question: ‘What works for whom in what circumstances and in what respects, and how?’ [23]. Proponents of realist review argue that traditional methods of review often focus on examining the effectiveness of interventions (i.e., whether interventions work or not) without developing our understanding of how, why, and when they work (or not) [22, 24]. The realist review method fills this gap by applying a configurational lens to develop an explanatory theory of how, why, for whom, and in what circumstances interventions work [21, 22, 25]. It lends itself to a context (c) + mechanism (M) = outcome (O) configuration heuristic for explaning causation [21, 22]. While other forms of reviews may examine the context, mechanism and outcome elements of interventions in isolation, a realist synthesis applies configurational thinking to explore their interface [22].

Realist review is underpinned by the idea that programmes or interventions are theories [22, 26, 27]. The configurational approach used by realists enables a more reflexive and iterative examination and illustration of programme theories underlying complex interventions [27–29]. Therefore, the unit of analysis in realist reviews is programme theory, but a C + M = O configuration heuristic is commonly applied to guide data analyses and syntheses. This means that evidence is analysed and synthesised to produce causal explanations for why, how, for whom and in what circumstances outcomes come about. Thus, realist reviews are configurational evidence syntheses as opposed to the aggregative nature of synthesis in conventional systematic reviews. This review follows five iterative steps recommended by various realist scholars: (1) defining the review scope; (2) developing initial programme theories; (3) searching for evidence; (4) selecting and appraising evidence; and (5) extracting, analysing and synthesising evidence [21, 22, 26, 30, 31].

### Defining the review scope

We first conducted a preliminary search of the literature with the aim of obtaining an overview of the literature on peer support in healthcare broadly and MCH in particular. We reviewed two categories of evidence retrieved from Google Scholar in March 2021: (1) empirical papers published between 2020 and 2021; and (2) review/conceptual papers published anytime. We used the search theme *‘peer support in healthcare’*. The search was informal and not based on structured selection criteria as we aimed to undertake an exploratory review of the literature on peer support in healthcare. We progressively reviewed the most current and conceptual papers retrieved to understand the range of candidate theories of peer support in healthcare broadly and develop a framework for our substantive review. The preliminary search guided our substantive review in the following ways: (1) defining the review questions and scope; (2) identifying initial (candidate) programme theories; (3) developing a literature search, appraisal and selection strategy for the substantive review; and (4) conceptualising the data extraction and synthesis framework. The realist review questions that emerged from the preliminary search were:

1. What are the causal *mechanisms* (‘why?’) that explain the effects of peer support interventions on MCH outcomes in SSA?
2. How do the characteristics of peers and intervention target group (‘for whom’) influence these *mechanisms and resulting outcomes*?
3. What is the influence of *contextual* factors (‘what circumstances’) on these *mechanisms*?

We draw on Pratley’s concept of MCH as encompassing ‘care utilization (obstetric care, ante- and/or perinatal care, delivery in a health facility), reproductive behaviours (utilization of modern contraceptives, birth spacing and ideal family size and/or number of children), women's health outcomes (anaemia, nutritional status and exposure to violence) and child health outcomes (nutritional status, diarrhoea, immunization status and lower respiratory infections)’ [32]. We look at the physical, mental and social well-being of mothers and children across the wide range of promotive, preventive, curative, rehabilitative and palliative health services they need [33]. This aligns with the broad range of indicators contained in the United Nation’s SDGs 3.1 and 3.2 that serve as measures of improvements in maternal and child health outcomes by 2030.

We operationalise context, mechanism and outcomes in this review as follows. Context here refers to the salient conditions within which peer support interventions in MCH in SSA are embedded. We analyse contexts at two levels: micro and macro. We consider the micro context as the characteristics of peers and intervention target groups, such as their demographic structures and norms. We consider the macro context as the broader institutional structure (environment) within which the groups and interventions operate. Examples include the wider national health policy and socio-cultural settings of specific groups and their interventions. Analysing the micro and macro contexts enables us to distil specific features of individual interventions alongside broader political, social, cultural and economic factors in different countries. We suggest that doing so allows the generation of findings, conclusions and recommendations that have both theoretical and practical implications beyond the SSA setting.

Given that realist research ‘begins with the researcher positing the potential processes through which a programme may work as a prelude to testing them’ [23], mechanism is considered the pivot around which realist research revolves [22, 26]. We use mechanisms here in relation to the underlying processes or ways by which peer support interventions bring about intended/unintended outcomes in maternal and child healthcare in SSA [21, 23, 24]. We examine and illustrate how and why various mechanisms are indicative of outcome patterns across different MCH services (e.g., breastfeeding, family planning, antenatal care and postnatal care) and SSA countries.

A fundamental feature of a realist synthesis is that ‘it is able to explain the complex signature of outcomes’ [23]. As discussed above, peer support groups naturally operate in different contexts and are underpinned by varied mechanisms. This suggests that the patterns of outcomes characterising different interventions may vary according to their underlying contexts and mechanisms. In health policy analysis, outcomes represent improvements, changes or impacts for targeted individuals, groups or populations attributable to the intervention under study [32, 34, 35]. In this review, we refer to outcomes as the intended and unintended results or impacts of peer support interventions. Specifically, we examine the nature and form of outcomes that different interventions generate at two levels: organisational and societal. By organisational level outcomes, we look at the improvements in the capacity and functionality of national health systems and healthcare organisations/providers to deliver MCH attributable to the intervention. We conceptualise societal-level outcomes to include the intended and unintended impacts of MCH peer support interventions for targeted individuals and groups or populations.

### Developing initial programme theories

Realist reviews often involve identifying initial (candidate) theories that potentially explain the CMO configuration of the intervention under study. They then proceed to ‘interrogate the existing evidence to find out whether and where these theories are pertinent and productive’ [21]. A programme theory is an overarching framework of how interventions are expected to work and what outcomes they are anticipated to create [22, 23]. The sources for eliciting initial programme theories are varied, namely, ‘documents, programme architects, practitioners, previous evaluation studies and social science literature’ [23]. Programme theories are often unique to individual settings or interventions containing well-defined metrics of outcomes against which the programme could be evaluated [23]. However, the intervention under review in this article falls into this category as it involves peer support across different areas of MCH within the diverse national/local social structures of SSA countries. In such complex realist research contexts, the initial programme theory can be one that is rooted in the theoretical literature [30, 36, 37]. Pawson and Tilley refer to this approach to elicitation of initial programme theory as drawing on ‘substantive theory’ [23].

**Fig. 1:**
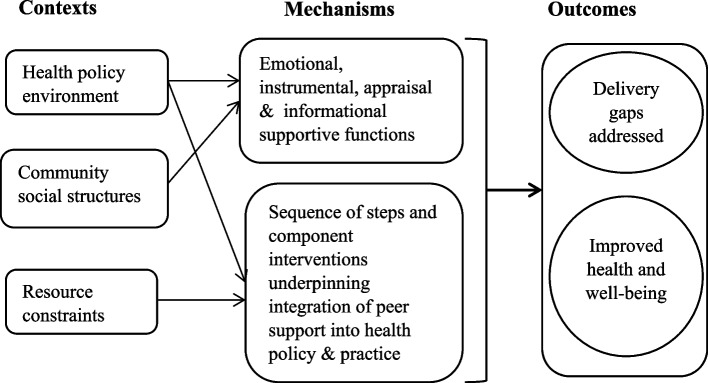
Initial programme theories of MCH peer support interventions

Drawing on the understanding that substantive theories represent ‘well-established theories within a particular field that help to explain why things happen the way they do’ [38], we based our elicitation of initial programme theories from our exploratory review of the broader literature on peer support. While exploring the literature on peer support in healthcare retrieved through a Google Scholar search conducted in March 2021 (see further explanation in “Defining the review scope” section), we paid attention to how scholars have attempted to hypothesise the realist question of ‘What works for whom in what circumstances and in what respects, and how?’ [23] (i.e., CMO configurations) in the broader discussion of peer support as interventions in healthcare contexts. We found two overarching programme theories with potential explanatory value for MCH peer support interventions in SSA: social support and resource-based theories. Figure 1 shows the hypothesised CMO configurations of these initial programme theories.

Social support theory is perhaps the most dominant programme theory of how peer support interventions are thought to promote improvement in health outcomes. The underlying principles of social support theory centre on the availability of social relationships for promoting health and well-being among people and communities [39–41]. This is implicit in both conceptual [10–14, 42] and empirical [43–46] literature on peer support in healthcare. Scholars frequently link peer support in healthcare to improved health and well-being attainable through four main mechanisms, namely, emotional, instrumental, appraisal and informational supportive functions [8, 40]. For example, peer support interventions are thought to promote emotional health through acceptance, encouragement and compassion [11, 47], instrumental support like financial assistance [8, 40], and informational support including the sharing of useful guidance and advice that facilitate healthy behaviours such as the appropriate use of drugs [6, 45].

The hypothesis is that peer support promotes health and well-being (outcome) through emotional, instrumental, appraisal and informational supportive functions (mechanisms). The fundamental context underlying this hypothesis is the availability of social support established by health policy and local community social structures. The hypothetical view is that the availability of peer support is sufficient for improved health and well-being to happen. This relates closely to the Ubuntu philosophy of Africa. Ubuntu is an African philosophy or ethic that views and approaches life and all its social dimensions like MCH, more as shared rather than individualistic citizenship [48–50]. Our focus on SSA, therefore, reveals an interesting opportunity to examine whether/how the prevalence of peer support may be shaped by indigenous socio-cultural contexts like the Ubuntu philosophy. This enables us to explore a quality dimension of the social support hypothesis. For instance, what contexts and mechanisms are most positive in promoting the health and well-being of mothers and children? As we highlight in “Defining the review scope” section, addressing this question requires exploring multiple dimensions of contexts (across micro and macro levels) and mechanisms (component interventions and sequence of steps).

The literature also reveals a resource-based theory of peer support in healthcare. For instance, Kåks and Målqvist [14] suggest that peer support interventions in high-income countries are complementary pathways to bridging access and coverage gaps. The overall view is that due to financial and human resource constraints, policy-makers and state-led healthcare organisations are increasingly integrating peer support groups into national health policy and service delivery programmes [11, 42]. Accordingly, the use of peer support groups in health service delivery helps to save governments the cost of wages and compensation that would be required to employ skilled health personnel. Additionally, peer support groups are seen as resources for filling staffing gaps in health service delivery [13, 42, 51, 52]. It is argued that the growing turnover of skilled health workforce globally presents peer support as a complementary source of lay/voluntary human resources for various health services [12, 13, 45]. Therefore, the hypothesis is that the integration of peer support interventions into national health policy and practice (mechanism) is considered to provide complementary financial and human resources needed to address delivery gaps (outcome) arising from resource constraints on governments and health systems (context).

The above hypothesis resonates with resource-based theories about the possession, utilisation and exchange of resources in classical economics [53, 54], management studies [55, 56], social psychology [57, 58] and sociology literature [59, 60]. Broadly, the resource-based theory in management studies and the social sciences generally proposes that firms engage in resource dependence, exchange or complementarity because they lack the needed resources to grow and become competitive [61, 62]. Given persistently limited budgetary allocations for health services and general fiscal constraints in SSA [63], critical analyses of emerging evidence on peer support interventions are needed to enhance our understanding of operational questions on (1) how to effectively integrate peer support resources into mainstream national health systems; (2) how to adapt the integration to local health priorities, given differences in social contexts within countries; and (3) how to sustain the integration.

This exploratory theorising is intended to highlight candidate programme theories that will be expanded during the iterative cycle of theory generation and refinement. Findings from the empirical review, brainstorming by the review team (via virtual meetings, phone calls and email discussions, stakeholders workshop with experts and practitioners and insights from a conference presentation (see “Refining the initial programme theory” section) will be used to refine the initial programme theory. By drawing on substantive theory to elicit our initial programme theory, the refined theory underlying MCH peer support interventions in SSA can be transferable to other health services or geographic settings.

### Searching for evidence

Following the preliminary search described in “Defining the review scope” section, we purposively conducted a database search for relevant studies using a three-heading search criterion: (1) mechanism–peer support; (2) context–sub-Saharan Africa; and (3) outcome–maternal and child healthcare. Four databases–Cochrane Library, PubMed, CINAHL, and EMBASE–were searched between 10th March and 3rd June 2021. The search strategy involved various combinations of terms related to the three-heading search criterion. Search entries and word truncations were based on the requirements of each database (see Additional file 1 for the search entries and outputs of each database). The search was limited to studies published in the English language.

**Fig. 2:**
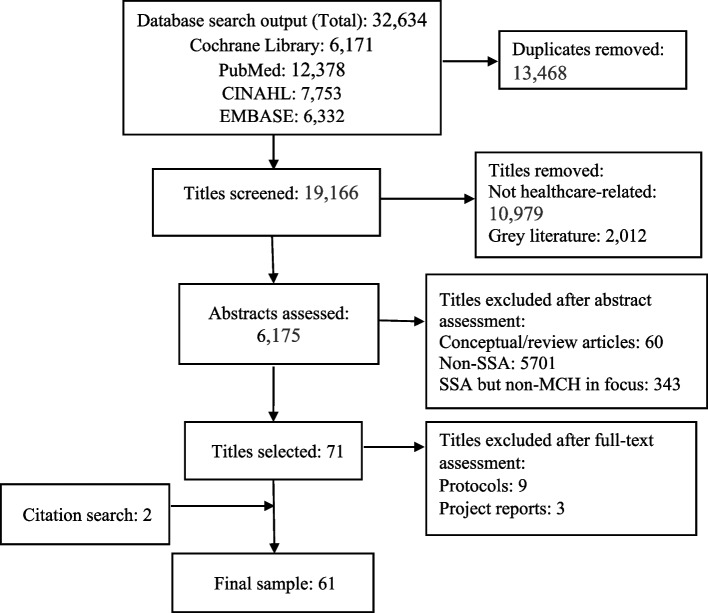
PRISMA flow diagram of literature search and appraisal process

The initial search generated a large volume of records. We also manually searched the reference lists of the studies selected for inclusion and some identified systematic review papers for additional evidence. We used the Preferred Reporting Items for Systematic Reviews and Meta-Analysis (PRISMA) flowchart to depict the number of records identified, included and excluded (see Fig. 2). In fulfilment of requirements to address the development of review protocols, we have attached a PRISMA-P checklist in Additional file 2. We will conduct a second search covering June 2021 to the present using the procedure described above before the final extraction and synthesis.

### Selecting and appraising evidence

We followed iterative steps to filter and appraise the evidence generated from the search so far, as summarised in Fig. 2. First, GD imported records from the database search into Endnote × 9 reference management software. Then, he removed duplicates using the software’s ‘find duplicates’ tool. Second, to ensure consistency, only GD screened the remaining records according to the inclusion and exclusion criteria. Following discussions within the review team, we considered only peer-reviewed articles on primary studies for inclusion in the review. There were no restrictions on the type of research design and publication date. GD manually screened 19,166 titles and excluded papers that were either not healthcare-related (10,979 titles) or were grey literature (2,012) such as blogs, conference presentations, dissertations, government documents and reports of ongoing interventions. Subsequently, the remaining 6175 abstracts were screened and records removed that were conceptual/review articles (60 titles), were conducted in other settings than SSA (5,701 titles) or did not focus on MCH (343 titles).

At the full-text screening stage, 13 articles were removed, including study protocols (9) and reports of ongoing MCH peer support projects (3) (see Additional file 3 for the list of screened studies). GD then applied the ‘relevance criteria’ recommended for realist review. Relevance thinking draws on the idea that the review team may limit their inclusion/ exclusion criteria to evidence containing data that is of use to the programme theory under development [25, 64, 65]. In traditional systematic reviews, researchers evaluate the quality of identified studies using structured appraisal tools like Evidence-Based Management guidelines, the Cochrane risk-of-bias tool and the Quality Appraisal of Reliability Studies checklist. A fundamental limitation of such a one-size-fits-all approach is the potential to exclude relevant evidence [66]. Through discussions within the review team, we agreed on the final list of selected studies based on two assessment questions: ‘Does the research address the theory under test?’ [22] and ‘Is this study good enough to provide evidence that will contribute to the synthesis?’ [25]. All 61 papers selected from the first search were found to be relevant. We will apply the same selection criteria in our planned second search.

### Extracting, analysing and synthesising evidence

#### Data extraction and initial coding

The extraction and synthesis of our final set of selected studies will be conducted in an iterative process described below. Two reviewers (GD and JA) will independently read and code the data. To ensure consistency, JA will concentrate on extracting a range of the study characteristics of the selected articles using a predesigned Microsoft Excel spreadsheet. This includes authorship, publication date, study setting, scope, aims, design and methods. GD will import papers selected for the review into the NVivo 12 software and then undertake repeated and close reading of the papers to code the data.

To develop an in-depth understanding of the different categories of contexts, mechanisms and outcomes, data will be coded under three parent nodes: context, mechanism and outcome. Child nodes (themes) under each parent note will be the basic categories of the contexts, mechanisms and outcomes across different MCH services and SSA countries. This enables us to develop an overview of the lists of contexts, mechanisms and outcomes reported in the empirical literature selected for review. In line with our realist lens, coding from the onset will be based on keywords, metaphors and lines of reasoning that address the review questions [67]. This involves close reading of the full text to identify themes from codes and realign emergent themes as the extraction progresses using the node hierarchies in NVivo (i.e., ‘parent’ and ‘child’ nodes). At the end of the data extraction and coding, a codebook will be generated, which will be reviewed independently by all members of the review team to ensure patterns occurring in the data are not missed.

#### Elicitation of CMO configurations and programme theory

Realist reviewers use retroduction to formulate CMO configurations and elicit programme theories. ‘Retroduction refers to the identification of hidden causal forces that lie behind identified patterns or changes in those patterns’ [68]. The fundamental question guiding realist reviewers’ elicitation of CMO configurations and programme theories is about the causal powers of the intervention within the given contexts in which it is developed and implemented. In this case, how is it that peer support intervention for different MCH services can produce observable outcomes in given conditions? Elicitation of CMO configurations and programme theories will be based on our three specific review questions:

1. What are the causal *mechanisms* (‘why?’) that explain the effects of peer support interventions on MCH outcomes in SSA?
2. How do the characteristics of peers and intervention target group (‘for whom’) influence these *mechanisms and resulting outcomes*?
3. What is the influence of *contextual* factors (‘what circumstances’) on these *mechanisms*?

Memo writing is considered an effective tool for carefully constructing theoretical arguments in realist research [69, 70]. ‘Memos are informal analytic notes about the data and the theoretical connections between categories [in this case context-mechanism-outcome]’ [71]. They represent the researchers’ thoughts and interpretations of the developing theory [67]. During this initial coding, GD will write memos to document ideas and thoughts on the CMO configurations and programme theories emerging from the evidence. This will involve annotating and taking notes of observed configurations and their underlying programme theories and examples of the contexts, mechanisms and outcomes illustrating them. Memo writing will follow an iterative process as the data analysis proceeds; initial ideas and thoughts of CMO configurations and programme theories will be revised as new patterns and insights emerge from further analysis of the evidence.

A memo will be written for every observable CMO configuration and its underlying programme theory found in the data. The memos will define the specific outcomes of MCH peer support interventions and the contexts and mechanisms driving them. Each observable CMO configuration will then be linked to the relevant initial programme theory defined in “Developing initial programme theories” section. If the configuration projects a new programme theory, the memo will highlight how this potentially provides grounds for refuting or refining the initial programme theories. Each memo will be linked to the source document(s) informing its development to allow other members of the review team to check.

The codebook generated alongside the memos written during the initial coding of data will be independently reviewed by the review team members. We will then hold several rounds of discussions to brainstorm and agree on the CMO configuration themes and related programme theories, which will be further refined through the steps detailed in “Refining the initial programme theory” section. The discussions leading to the development of CMO configuration themes and related programme theories will pay attention to projecting a constant comparative analysis of the generative causality between the different MCH services and national settings.

#### Refining the initial programme theory

Realist scholars believe that the question about the causal powers of interventions within given contexts cannot be adequately addressed by using only observable evidence [68, 72]. We will combine the views and experiences of practitioners and the interpretations and expertise of scholars to cross-examine, support, refute, or refine CMO configurations and programme theories emerging from the evidence reviewed [27, 72]. This approach aligns with the realists’ view that establishing multiple sources of reasoning to develop programme theories–such as intervention elements, participant responses, interpretation of the original researcher or interpretation of reviewers–is crucial for demonstrating theoretical relevance and rigour [22, 28]. Specifically, we will refine the initial programme theories and their take-up in the reviewed literature through three steps [26].

First, we will convene a stakeholders’ workshop in Ghana to discuss our findings with and generate inputs from a wide range of experts including policy-makers, health directors, representatives of peer support groups and civil society organisations, and nurses/midwives. This will provide an opportunity to compare the emerging programme theory and the experiences of practitioners and policy-makers. Further details on the stakeholders and the workshop will be included in the final review for readers to check. Second, we will present our findings at an international conference which will enable us to generate fresh ideas and suggestions for further refinement of the programme theory. Third, the review team will discuss the refined programme theory emerging from the previous steps and link it back to the substantive theory in the existing literature described in the “Developing initial programme theories” section. The final output of the review will be a more refined programme theory with explanatory value for peer support interventions beyond MCH and SSA contexts.

## Discussion

Our focus on SSA resonates with emerging calls from scholars and policy-makers for attention to social and institutional peculiarities in developing and evaluating peer support interventions [9, 11, 15]. These calls for attention to context require a review method that systematically explains ‘what works for whom, in what circumstances, in what respects and how’ [22]. Using realist review methodology enables us to examine the context-mechanism-outcome configurations of different forms of MCH peer support interventions in SSA. Given the large volume of literature on peer support in healthcare, we concentrate on MCH to achieve an in-depth synthesis of evidence and explication of theory. In reviewing the literature on different forms of MCH, we consider our review to provide generalizable practical and theoretical lessons on peer support interventions in other health services. As explained in previous sections, persistent adverse outcomes and access gaps peculiar to MCH in resource-constrained settings like SSA further justify our review focus.

The review is considered to articulate an explicit programme theory of peer support intervention in healthcare delivery and provide insights for developing evidence-informed policy on the intervention. First, by drawing lessons from the different national contexts and diverse areas of MCH, our review has the potential to provide a generalizable programme theory that can guide intervention design and implementation. While focusing on SSA, we aim to contribute to evolving conversations on the use of theory for health policy planning and intervention design and implementation globally. Second, we hope the review identifies practical ways of developing effective and sustainable peer support initiatives. We expect our findings to highlight some latent and unique properties of the SSA story, which we term reverse innovation [73], that health policy-makers and leaders in non-SSA settings like high-income countries may draw on to develop creative peer support interventions.

### Supplementary Information

**Additional file 1.**

**Additional file 2.**

**Additional file 3.**

## Data Availability

All data generated or analysed in this review are included in this article and its supplementary information files.

## Abbreviations

CMO: Context-Mechanism-Outcome
HIV: Human Immunodeficiency Virus
MCH: Maternal and Child Healthcare
MDGs: Millennium Development Goals
PRISMA: Preferred Reporting Items for Systematic Reviews and Meta-Analysis
PRISMA-P: Preferred Reporting Items for Systematic Reviews and Meta-Analysis Protocols
SDGs: Sustainable Development Goals
SSA: Sub-Saharan Africa
WHO: World Health Organization
