# Supplementary material for: Peer support interventions in maternal and child healthcare delivery in sub-Saharan Africa: protocol for a realist review
Source: Syst Rev. 2023 Oct 25;12:199. doi: 10.1186/s13643-023-02366-3 (PMC10598906; doi:10.1186/s13643-023-02366-3)
Supplement: Supplementary file 1 — Additional file 1. [file 13643_2023_2366_MOESM1_ESM.pdf]

Appendix 1: Database search entries and outputs

Search	Database	Records found
**Cochrane Library**		
#1	(“peer support*”) OR (“peer counsel*) OR (“peer mentor*”) OR (“peer led”) OR (“mother to mother support*”) OR (“mother-to-mother support”) OR (“M2M”) OR (“expert mother”) OR (“mentor mother”) OR (“mother club”)	2,239
#2	(“maternal health”) OR (“women health”) OR (“maternal, newborn and child health”) OR (“antenatal care”) OR (“pregnancy care”) OR (postnatal care”) OR (“child health”) OR (“infant feed*”) OR (“breastfeed*”)	18,905
#3	(“Africa”) OR (“sub-Saharan Africa”) OR (“low and middle income count*”) OR (“developing count*”)	13,052
#4	#1 AND #2 AND #3<br>(limited to only Cochrane Reviews –238 and Trials – 5933)	6,171
**PubMed Central**		
#1	(“peer support” OR “peer counsel*” OR “peer mentor*” OR “peer led” OR “mother to mother support” OR “mother-to-mother support” OR “M2M” OR “expert mother” OR “mentor mother” OR “mother club”) AND (“maternal health” OR “women health” OR “maternal, newborn and child health” OR “antenatal care” OR “pregnancy care” OR “postnatal care” OR “child health” OR “infant feed*” OR “breast feed*”) AND (“Africa” OR “sub-Saharan Africa” OR “low and middle income count*” OR “developing count*”)	12,378
**CINAHL (EBSCO)**		
#1	("peer support" OR "peer counsel*" OR "peer mentor*" OR "peer led" OR “mother to mother support” OR "mentor mother") AND ("maternal health" OR “maternal, newborn and child health” OR "breastfeed*") AND ("sub-Saharan Africa" OR “low and middle income count*” OR "developing count*")	7,753
**EMBASE**		
#1	(“peer support” OR “peer counsel*” OR “peer mentor*” OR “peer led” OR “mother to mother support” OR “mother-to-mother support” OR “M2M” OR “expert mother” OR “mentor mother” OR “mother club”)	2,814
#2	(“maternal health” OR “women health” OR “maternal, newborn and child health” OR “antenatal care” OR “pregnancy care” OR “postnatal care” OR “child health” OR “infant feed*” OR “breast feed*”)	19,832
#3	(“Africa” OR “sub-Saharan Africa” OR “low and middle income count*” OR “developing count*”)	66,643
#4	#1 AND #2 AND #3	

#5	(“peer support” OR “peer counsel*” OR “peer mentor*” OR “peer led” OR “mother to mother support” OR “mother-to-mother support” OR “M2M” OR “expert mother” OR “mentor mother” OR “mother club”) AND (“maternal health” OR “women health” OR “maternal, newborn and child health” OR “antenatal care” OR “pregnancy care” OR “postnatal care” OR “child health” OR “infant feed*” OR “breast feed*”) AND (“Africa” OR “sub-Saharan Africa” OR “low and middle income count*” OR “developing count*”)	6,331
